# Encephalopathy Due to COVID-19 With Great Response to Glucocorticoids

**DOI:** 10.7759/cureus.17845

**Published:** 2021-09-09

**Authors:** Lucca Pizzato Tondo, Eduardo Beck Paglioli Neto, Soel Arpini, Giordani Passos, Jefferson Becker

**Affiliations:** 1 Neurology, Pontifical Catholic University of Rio Grande do Sul (PUCRS), Porto Alegre, BRA

**Keywords:** covid-19, covid 19 encephalitis, encephalitis, corticoid, covid-19 treatment, encephalitis treatment, mild to moderate covid-19

## Abstract

Minor neurological symptoms such as anosmia are relatively common manifestations of coronavirus disease 2019 (COVID-19). However, severe affection of the central nervous system (CNS) occurs in a minority of cases and its treatment and pathophysiology is not yet well understood. It has been described that encephalitis due to COVID-19 may be caused by the proinflammatory state due to the cytokine storm or direct invasion of the virus in the CNS. Here we present a case of a 66-year-old man with bipolar disorder and moderate respiratory COVID-19 symptoms who presented to the emergency department with a decreased level of consciousness. Brain computerized tomography (CT) showed no acute pathology. A thorough investigation of other possible causes of CNS affection was negative. The patient was treated with pulse therapy with methylprednisolone and presented a significant improvement of his neurological condition, being discharged with a complete neurological recovery five days after the start of the treatment. This case illustrates the importance of a high index of suspicion in diagnosing severe CNS impairment in mild respiratory COVID-19 cases. Also, this case corroborates with previous reports of glucocorticoid response in CNS impairment associated with COVID-19, although more robust studies are required to confirm this relation.

## Introduction

The coronavirus disease 2019 (COVID-19) pandemic that has hit the world since the end of 2019 has now caused more than 180 million cases of infection around the globe according to the World Health Organization [1]. Severe acute respiratory syndrome coronavirus-2 (SARS-CoV-2) uses the angiotensin-converting enzyme 2 (ACE2) receptor to invade the human cells and lead to the infection that commonly causes manifestations including fever, cough and fatigue [2]. Although the major clinical presentation of COVID-19 infection comprises the respiratory tract, other manifestations are being increasingly reported [3]. Neurological symptoms are not rare, especially in patients with chronic medical conditions [4], and it is important for healthcare professionals to be aware of them [2]. Here we report a case of encephalopathy due to COVID-19 with great response to glucocorticoids.

## Case presentation

**Figure 1:**
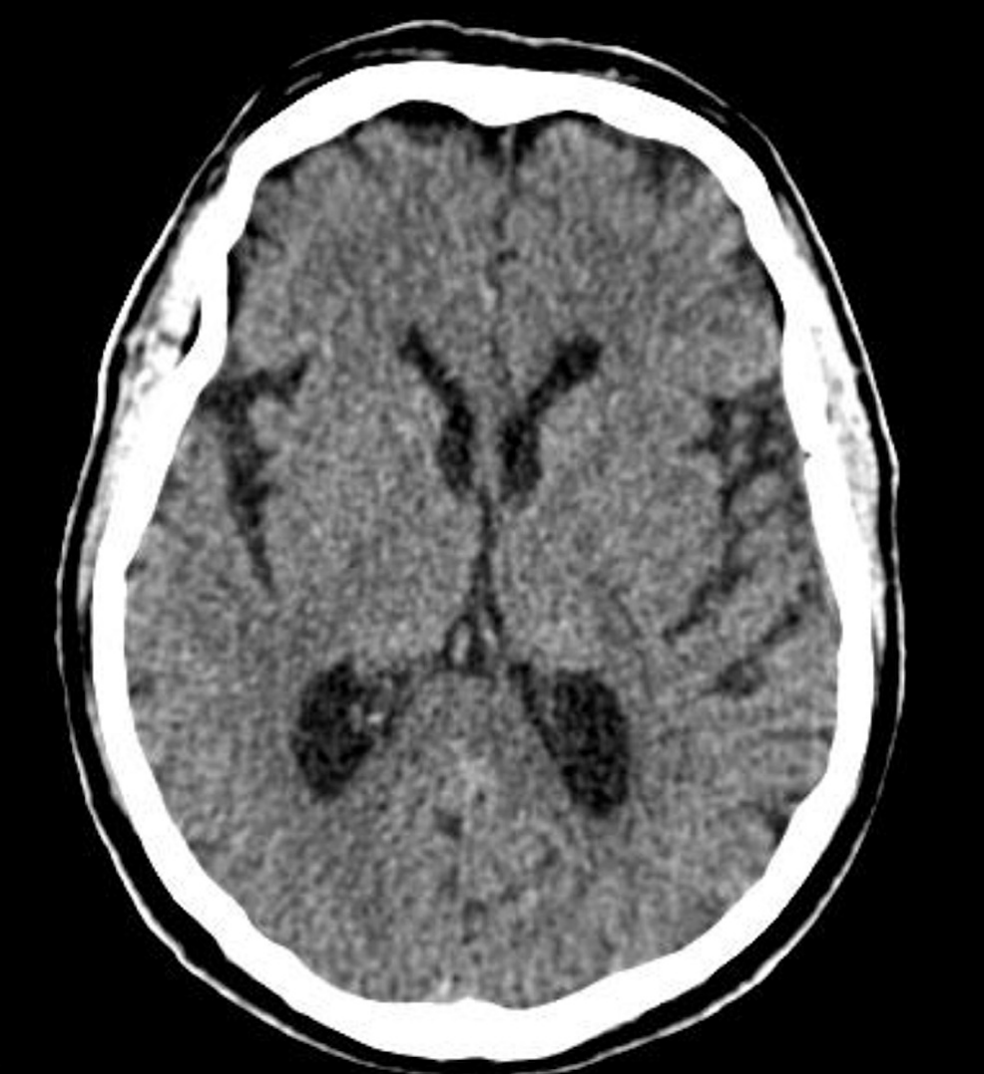
Computerized tomography (CT) of the head showing moderate parenchymal atrophy and microangiopathy. There was no sign of any acute pathology.

A 66-year-old male with bipolar disorder and hypertension presented to the Emergency Room due to decreased level of consciousness 14 days after a COVID-19 diagnosis through real-time reverse-transcriptase polymerase chain reaction. He had been discharged from the hospital two days ago due to an atrial flutter and mild respiratory COVID-19 symptoms, with lung imaging showing 10-20% of ground-glass opacities. Vital signs at the present admission were 84 beats per minute with a sinus rhythm, blood pressure of 128/80 mmHg, and 94% oxygen saturation on room air. On physical examination, the patient was afebrile, with a Glasgow Coma Scale of 14, not oriented in time and space, with hyperreflexia and globally decreased strength. He presented no signs of meningism, no abnormal gait, no focal deficit, and no signs of respiratory distress nor hemodynamic instability. Laboratory testing revealed leukocytosis with normal differential, normal electrolytes, normal d-dimers, C-reactive protein of 5.9 mg/dl, creatinine of 3.49 mg/dl, urea of 175 mg/dl and serum lithium of 2.3 mEq/L, which was suspected to be the cause of his obnubilation. Brain computerized tomography (CT) only showed signs of microangiopathy, with no acute process explaining his mental state alteration (Figure 1).

**Figure 2:**
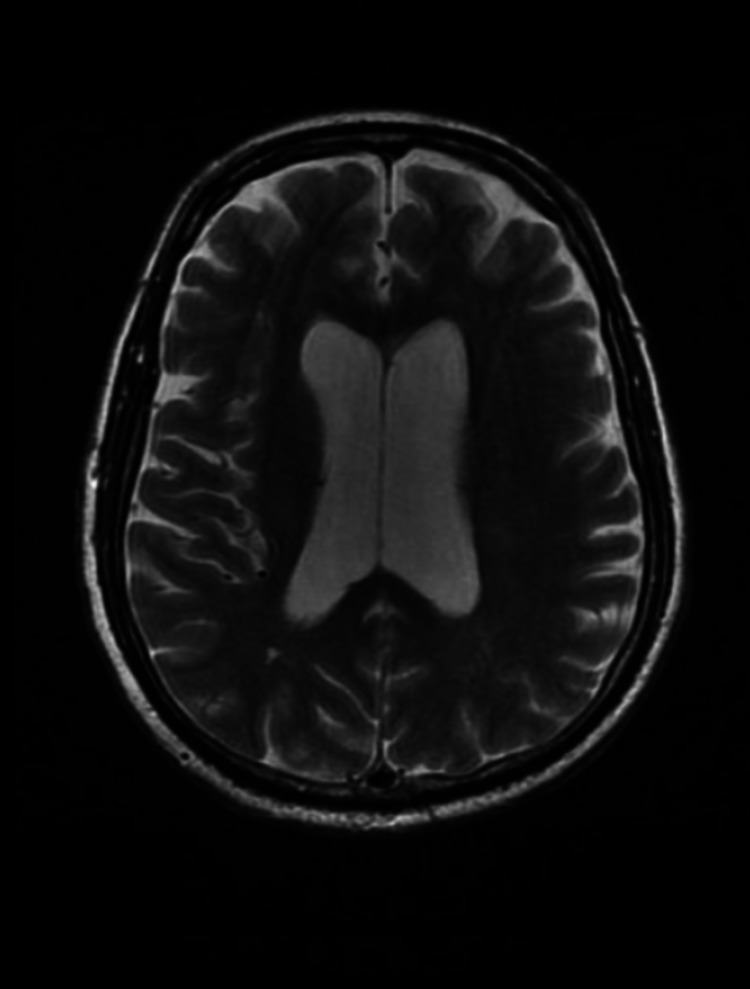
Axial T2 magnetic resonance imaging (MRI) showing ventricular mild ectasia.

He was admitted to the hospital for further investigation and lumbar puncture was postponed due to the fact that the patient was taking anticoagulants because of his recent episode of arrhythmia. Lithium was suspended as it was hypothesized that his altered mental state could be caused by lithium intoxication. Despite his improvement of renal function and serum lithium level on the following days, the patient presented a worsening of his neurological status. His Glasgow Coma Scale decreased to 11 and he presented myoclonic movements in upper and lower limbs. Further investigation revealed a suppressed thyroid stimulating hormone (TSH), with normal anti-thyroid peroxidase (TPO), T3 and T4. Cerebrospinal fluid (CSF) showed no evidence of infection in the central nervous system (CNS). CSF analysis was clean, with glucose of 85 mg/dl, proteins of 10 mg/dl and normal leukocytes with predominant mononuclear cells. CSF testing was negative for tuberculosis, cryptococcosis and syphilis. There were identical IgG oligoclonal bands in CSF and plasma serum indicated systemic inflammation. Electroencephalogram (EEG) showed moderate cortical encephalopathy. Magnetic resonance imaging (MRI) revealed discrete ectasia of the supratentorial ventricular system, associated with slight erasure of the paramedian brain gyri, which may be associated with some degree of normal pressure hydrocephalus and also mild to moderate cerebral volumetric reduction (Figure 2). Furthermore, in the T2 and T2/fluid-attenuated inversion recovery (FLAIR) sequences, there were rare foci of hypersignal in the periventricular and deep white substance, related to incipient microangiopathy.

By the fifth day after admission, the patient was hemodynamically stable, recovered his renal function and was breathing comfortably in room air. Despite the improvement of his general condition, his neurological status kept declining. Considering that other causes of primary CNS affection (such as meningitis, fungi infection, tuberculosis, autoimmune encephalitis and syphilis) were excluded, it was hypothesized that the patient’s impaired neurological status was due to a COVID-19-related encephalopathy. Based on previous studies reporting improvement of neurological condition of COVID-19 patients after corticosteroid treatment, pulse therapy with methylprednisolone 500 mg IV for three days was started on day five after admission. A significant improvement in his neurological condition was seen on the first day of therapy. He was discharged five days after the start of methylprednisolone with a complete recovery of his mental status. In an outpatient consult two weeks post-discharge, the patient remained with significant neurological improvement, but complaining of transitory aphasias and lapses of memory, happening three times a week and lasting for a few seconds. His new EEG had no abnormalities.

## Discussion

Encephalopathy is a pathobiological process in the brain that usually develops over hours to days and can manifest as changed personality, behaviour, cognition, or consciousness [5]. Although some extent of neurological symptoms is seen in over 80% of COVID-19 patients [6], critical CNS conditions such as encephalopathy and encephalitis are rarer conditions that occur more frequently in patients with severe COVID-19 [4]. The prevalence of encephalopathy associated with COVID-19 is highly variable in the literature, ranging from 7 to 69% [3,4,6]. However, such a condition is mostly associated with hypoxic and metabolic changes in severe COVID-19 cases [4]. In that sense, the patient presented in this case was different from the expected. He presented mild respiratory COVID-19 symptoms not requiring invasive ventilation and no systemic impairment that could explain his CNS impairment.

There are multiple and independent theoretical mechanisms implicated in neurological impairment in COVID-19. These include direct virus invasion, systemic dysfunction due to cardiorespiratory distress and proinflammatory state with cytokine storm [7]. Neuroimaging finding includes unknown origin encephalitis with negative neuroimaging findings, acute disseminated encephalomyelitis, acute necrotizing encephalopathy, limbic encephalitis and nonspecific alterations [8]. Also, endotheliitis of the posterior circulation was reported in patients with high significant response to glucocorticoids [9]. Our patient presented discreet ectasia of the supratentorial ventricular system, mild cerebral volumetric reduction and rare foci of hypersignal in the periventricular and deep white substance.

Diagnostic criteria for COVID-19-related encephalopathy are not well established in the literature [10]. Possible encephalopathy can be suspected in patients with SARS-CoV-2 detected in respiratory or other non-CNS samples and no other explanatory pathogen or cause for CNS affection [3]. Lumbar puncture can be normal or reveal protein increase and pleocytosis [4], and SARS-COV2 could be isolated only in a minority of cases [8]. Most studies report EEG with nonspecific changes and near-physiological patterns with scarce abnormalities [4]. The diagnosis of COVID-19-related encephalopathy was given to our patient based on the fact that his neurological status declined despite the improvement of his general condition and other major causes of CNS affection were discarded.

Our patient presented a dramatic response to three-day intravenous methylprednisolone 500mg, being discharged from the hospital five days after the beginning of the pulse therapy without neurological abnormalities. Even though the MRI, CSF analysis and EEG had no characteristic findings, the immense response to steroid treatment and the exclusion of other causes supports SARS-CoV-2 encephalopathy theoretical pathophysiological mechanisms. Similar cases of COVID-19-related encephalopathy responsive to high-dose glucocorticoids are being reported around the globe [9,11]. Further studies and trials are needed to elucidate the real effectiveness and responsiveness of encephalopathy to corticosteroids [9].

## Conclusions

Encephalopathy is a potentially severe COVID-19 manifestation that is usually seen in severe presentations of the disease and is associated with great morbimortality. Because of its highly variable presentation, the diagnosis of encephalopathy related to COVID-19 requires a high index of suspicion. Here, we described a patient with mild COVID-19 symptoms who developed an encephalopathy highly responsive to glucocorticoids. This report complements the current literature on CNS affection due to COVID-19, and it corroborates with previous studies suggesting a potential role of glucocorticoids in the treatment of COVID-related CNS affection. More robust studies must be conducted to evaluate the best treatment in COVID-19 patients.
